# Precarious hope and reframing risk behavior from the ground up: insight from ethnographic research with Rwandan urban refugees in Yaoundé, Cameroon

**DOI:** 10.1186/s13031-019-0206-0

**Published:** 2019-05-22

**Authors:** Kelly Ann Yotebieng, Nathan Fakult, Paschal Kum Awah, Jennifer L. Syvertsen

**Affiliations:** 1 Ohio State University, Department of Anthropology, Columbus, OH USA; 2 Department of Anthropology, Faculty of Arts, Letters and Social Sciences, University of Yaoundé I, Yaoundé, Cameroon; 3 Centre for Population Studies and Health Promotion, Yaoundé, Cameroon; 4 Department of Anthropology, University of California, Riverside, USA

**Keywords:** Gender, Risk, Health behavior, Sex work, Non-compliance, Refugees, Cameroon

## Abstract

**Background:**

Theoretical and methodological research on risk-taking practices often frames risk as an individual choice. While risk does occur at individual level, it is determined by aspirations which are connected to others and society. For many displaced women globally, these aspirations are often linked to the well-being of their children and other household members. This article explores the links between aspirations for the future, gendered household dynamics, and health risk-taking behavior among the Rwandan urban refugee community.

**Methods:**

This analysis drew from participant observation, focus group discussions, and in-depth interviews with 49 male and 42 female household members from 36 Rwandan refugee households in Yaoundé, Cameroon. The fieldwork was conducted over 12 months between May–August 2016, May–August 2017, and February–August 2018.

**Results:**

We observed that while there was considerable convergence among household members in aspirations, there was considerable difference in risk-taking practices engaged to achieve them with women often assuming the greatest risks. These gendered realities of risk were not only related to structural concerns including access to different forms of capital, but also to socio-cultural gendered expectations of women, how risks were defined and justified, and household dynamics that drove the gendered reality of observed risk-behavior.

**Conclusions:**

Humanitarian programs and policies are distinctly finite in nature; focused on the short-term needs of persons affected by conflict. However, many humanitarian situations in the world are protracted. In the midst of these challenges, themes of future-orientation, possibilities, and shared aspirations for a better future emerge. These aspirations and the practices, including risk-taking practices that stem from them are central to understand if we are to ensure a just peace and stability in displaced communities throughout the developing world. Our analysis highlights the need to examine sociocultural dimensions related to hopes for the future, gender, and household dynamics as a way to understand risk behavior. We propose this can be done through a framework of precarious hope which we put forward in this paper, in which hope, agency, sociocultural and political economic contexts situate risk as a gendered practice of hope amidst constraint.

## Background

Risk is a ubiquitous focus of research across public health, medicine, and social science scholarship on refugees and displaced persons. Within public health, risk research focuses on risk perceptions, risk behavior, and environmental risk factors [1–4]. Risk behavior research in public health aims to understand the ways in which behaviors that may result in physical harm or higher probability of exposure to poor health outcomes can be better understood and mitigated. Public health research on risk behavior often consists of identifying statistically significant “risk groups” [5–7]. Among these, marginalized populations including refugees are often the focus of public health research on risk perception, risk behavior, and environmental risk factors that are associated with a number of health outcomes [8–11]. However, research has pointed out that risk behavior in its many forms is often not understood, justified, and undertaken by individuals engaging in these actions with the same underlying assumptions held within the public health community [12]. This article aims to further this conversation on how we understand and study risk behavior among women refugees by examining the social, cultural, and structural determinants of risk behavior, as well as the logic behind these actions and the ways they are linked with aspirations for the future. We do this using the case of Rwandan urban refugees in Cameroon. In this paper, we underline how socio-cultural contexts may also influence gender disparities in the ways that persons decide to engage in risk behavior. As our research participants never explicitly referred to their behavior as “risky,” we use the term “strategies” to examine these actions through the results and discussion section.

While this paper focuses primarily on a framework to better understand risk behavior, our findings also overlap with the broader literature on risk, including the risk perceptions and environmental risk factors that are inextricably linked with risk behavior. Risk and resilience are important concepts in the humanitarian paradigms that guide programs geared towards refugees and displaced persons. Risk perceptions, behavior, and environmental risk are essential components to understanding resilience. In many humanitarian resilience models, risk behavior is framed as choices that lead to unexpected, nonlinear better or worse outcomes [13–16]. However, Panter-Brick [17] eloquently puts forward an interdisciplinary path to improve resilience models by understanding the hope that drives risk. We posit that expanding our understanding of risk behavior from multiple perspectives and the relationship between these strategies and self-defined outcomes are critical to better develop strategies aimed at mitigating health risk behavior.

Much of the public health research on risk perception and behavior aims to understand risk as individualized choices [5–7, 18, 19]. Yet, risk perception is intricately linked with risk behavior, and other social scientists have long tried to frame and understand risk perception as a socio-cultural construct [20–24]. Douglas’ [20–22] theoretical research situated risk perception as a function of societal norms and individual perceptions of when these norms were transgressed. Douglas’ [20–22] work, foundational in the social science research on risk perception, underlines the ways in which individuals within societies perceive of risks within their own frame of references and the competing factors that are important to them. More recent research has revisited risk perception as a socio-cultural construct, underlining the ways in which what is perceived to be a risk and the efforts made to mitigate risk have changed over time, even within the field of public health [23, 24].

Other research focuses on environmental risk factors: linking risks to broader social ecological and political economic contexts, and often frames it as rooted in inequality [2, 4, 25, 26]. Giddens [26] underlines that agency is an essential component to understand the ways that environmental risk factors are both created and mitigated. This agency occurs within broader societal structures and access to various forms of capital [27, 28]. Differential access to various forms of capital affect a person’s experience in the world in terms of the spaces that they can easily navigate, and the ways in which they navigate these spaces. Appadurai [29] has astutely underlined however that limited access to capital does not mean that marginalized persons lose hope or cease to develop and work towards better futures. In this way, broader environmental risk factors may lead to the need to engage in higher risk behavior. As access to capital becomes more challenging, marginalized persons are forced to reassess the pathways towards their aspirations. These aspirations, framed within the broader socio-cultural and political economic contexts, become the motivating factors behind observed actions. This may lead to higher risk-taking practices among the most marginalized [30]. In this way, Rhodes [4] argues that individualistic behavior change models will be largely ineffective in mitigating risk behavior and optimizing public health outcomes.

Globally, refugees live in unequal political economic contexts that drive a multitude of environmental risk factors. This includes restricted access to different forms of capital driven by various forms of hardship including xenophobia and exclusionary immigration policy. In these broader environmental contexts it is increasingly difficult to navigate different social spaces, therefore decreasing options that could otherwise allow individuals to achieve their desired goals [29, 31, 32]. Access to capital, and therefore the prioritization of one’s aspirations in a household or society, is also affected by socio-cultural gendered realities [28]. In particular, women in contexts of increasing austerity may also feel that their aspirations and futures are of lesser value than their husband’s, brothers’, or children’s. Furthermore, in many contexts, including the Rwandan contexts, Rwandan women are expected to make sacrifices if it means ensuring their children’s well-being [33–35]. The fulfillment of these socio-cultural expectations are another important motivating factor towards actions engaged by persons. In this setting a focus on the ways in which marginalized persons resist social inequalities, and the consequences that are related to that resistance, can better help us understand what drives risk behavior [28].

### Research setting

This research focuses on Rwandan urban refugees in Yaoundé, Cameroon. Yaoundé is a growing urban center in a lower-middle income country experiencing both demographic transition and an influx of refugees and immigrants, allowing findings from this research to be applicable to other cities in Africa and the developing world. Yaoundé is estimated to be growing at a rate of 5% each year, with growth primarily occurring in areas already struggling with adequate access to electricity, water, sanitation, and other city services [36, 37]. Despite a large number of Cameroonians already struggling to meet their basic needs, the country continues to host growing numbers of refugees from surrounding countries [38–40]. According to the most recent published refugee statistics, at the end of 2017, there were an estimated 598,570 refugees, internally displaced persons, and asylum seekers in Cameroon, of which over 22,000 are estimated to live in urban areas [41].

Rwandan Hutu refugees began arriving in Yaoundé in 1994, and in larger numbers between 1996 and 1998 as they fled insecurity in refugee camps in Eastern Democratic Republic of Congo [39, 42]. In recent years, many Rwandan refugees are in danger of losing international protection, which their refugee status provides, under the purview of the UNHCR’s Cessation Clause [43]. While Cessation Clauses are not common, they have been invoked in other cases, mostly in Africa [38, 44–48]. Unwilling to return to Rwanda, the Rwandan urban refugee community feels that the loss of their legal status may be a tipping point, or something that can result in the need to engage in higher risk activities to sustain their households [49]. Using evidence from this 3-year ethnographic research project, in this paper we will propose a framework of precarious hope to improve our understanding of the factors driving strategies that are often framed in the public health literature as risk behavior among urban-based refugee populations. After all, the majority of the world’s refugee populations live in urban areas, often incurring enormous challenges to arrive and thrive in these settings [50].

## Methods

### Theoretical foundations

A forward-looking framework that examines strategies in the context of marginalized persons’ desired futures and self-defined outcomes, and the ways in which a focus on the future leads to a greater ability to tolerate uncertainty can be a productive way to reframe risk behavior [30]. To ensure a future orientation, we use Appadurai’s [29] capacity to aspire framework as a foundation for this research. The capacity to aspire framework considers the ability to imagine and act on aspirations as a shared cultural capacity situated firmly within a society’s context, structures, and unequal access to various forms of capital. The capacity to aspire cannot be divorced from the contexts of inequality in which marginalized persons find themselves. The capacity to aspire situates aspirations in concrete terms by underlining what it is a person wants to achieve (aspirations), a belief that they can be achieved (hope), the actual feasibility of any plan to achieve them (political economy), and the strategies taken to achieve them despite the challenges stacked up against them (practices) [29]. These aspirations become the primary motivating factors behind strategies observed in the present.

In this paper, we build on the capacity to aspire framework to develop a grounded theory [51] of precarious hope. The precarious hope framework, which we outline in the discussion, resituates our understanding of risk to demonstrate the ways in which aspirations and strategies interact, and the central role of gender in understanding these aspirations and strategies. To do this we used ethnographic methods to examine the reciprocal relationship between research participant’s aspirations, their ability to achieve them within their socio-cultural and gendered contexts, and the structural determinants affecting them.

### Household as the unit of analysis

In this research we observed sub-units or groupings (usually dyads or triads) of persons that emerged through participant observation within households as the unit of analysis. Intra-household dynamics within households determined decisions taken by individual household members, and aspirations were often shared among household members. From our observations, the household was the level at which explicit and implicit action towards aspirations is decided.

### Study design, population, and sampling

The study design [42, 43, 52] was qualitative using a longitudinal ethnographic research approach that principally relied on participant observation. The broader research project focused on the social determinants and constructions of health, wellbeing, and strategies among urban refugees. We identified research participants using a snowball sampling approach [53]. Participants were purposively sampled based on different lengths of time in country and different household compositions (e.g. female headed, orphan-headed). We first identified community leaders from our existing contacts in the field through our past work with urban refugees in Yaoundé. Yotebieng had conducted prior research and lived in Yaoundé, so was able to pull on her contacts for initial participants. Only 3 research participants were directly referred by refugee agencies where a service provider mentioned Yotebieng, and they reached out directly to her. Each individual and family invited Yotebieng via a snowball sampling approach to meet 1–2 other individuals within their social networks. Care was taken to make sure that no research participant referred more than 2 additional to ensure that the sample was comprised of a mix of persons who did and did not have contact with refugee serving agencies. We sampled multiple household members within a household for in-depth interviews, and conducted participant observation within households and with several members of each household in their daily activities outside of the home.

### Data collection and analysis

The data for this paper were collected via 12 months of fieldwork conductive over 3 years between May, 2016 and August, 2018 [42, 43, 52]. Yotebieng collected initial data through focus group discussions, daily participant observation, 30 semi-structured interviews, and further unstructured conversations that occurred during participant observation (see below) with research participants. All interviews were recorded and transcribed.

During observations and interviews the predominant language was French, with translations in Kinyarwanda provided with support from a translator during unstructured interviews on three occasions when it was necessary. Participant observation was documented through Yotebieng’s field notes (a total of 408 field notes were generated over the course of this research) in English and transcribed audio-recorded conversations in French.

Participant observation allowed us to connect responses to the semi-structured interview questions on aspirations to actions of household members by asking questions related to interview responses. Furthermore, it was a way to observe practices that were often not discussed during semi-structured interviews. Participant observation also allowed us to analyze the different strategies taken by different household members towards achieving their aspirations, and to observe some of the emotional aspects of aspirations and hope, including caretaking, that are not always readily discussed during in-depth interviews.

During participant observation sessions, Yotebieng engaged in continued informal discussions where she asked household members about their aspirations, or what they valued and were striving for in their lives. She also tried to understand their daily activities, and how these related in different ways to their aspirations. During these daily activities, she documented interactions, such as conversations, disagreements, or joint activities between household members. As trust was developed, she accompanied households and their members in daily activities outside the home, including participating in community meetings and events (churches, etc.). Yotebieng also observed and included in field notes differences in discourse within the household to understand the power-laden reality of discourse including who sets the tone, who defines the terms, and what is acceptable to think or do.

Yotebieng developed a comprehensive open coding scheme that was inductively derived from and applied to the field notes and transcripts using MaxQDA software in order to produce a fine-grained descriptive analysis [51–55]. Careful attention was paid to mention of characteristics related to the factors that research participants felt impacted their hopes and aspirations for the future, and the actions they were engaging in to address these challenges. Memos were included to draw attention to any remarkable events, justifications for the use of specific codes, and discussions of relationships emerging between codes.

Our data analysis procedures, guided by the principles of grounded theory [51], allow us to put forward an ethnographically informed theoretical explanation of the process that links aspirations to gendered disparities in practice. Pursuant to a grounded theory approach, we continuously analyzed the data and followed new leads that emerged related to our research question, as a way to build a rich explanation grounded in the real experiences and practices of the research participants. Pseudonyms are used to protect the confidentiality of our research participants.

## Results

Our research participants included 49 male and 42 female refugees from 36 households in the Rwandan community who Yotebieng spent significant time with during participant observation. All participants were older than 18, with ages ranging from 22 to 73 years old. The majority of heads of household were women (58%), with an average age of 44 years of the head of household (range: 23–72). All were currently living in Yaoundé. Most of the refugees had been living in Cameroon for a decade or more, but a smaller proportion had arrived more recently, fleeing insecurity in countries where they were previously resettled including the Central African Republic and the Republic of Congo. Every household reported at least one person who was struggling with long-term chronic illness including hypertension, diabetes, and HIV.

Economic pressure emerged strongly over the course of this research. The majority of the Rwandan urban refugee community in Yaoundé consisted of low income households led either by women, often widows, or adult sons. Their primary source of revenue was in the informal sector: hawking or working in small stores throughout slums scattered across the city. Many of these stores were either operating on credit, or the refugees were employees of Rwandan businessmen. Most of their houses did not have running water, and usually the sleeping conditions included at least three persons on a mattress on the ground in a corner of a main room separated from what became a makeshift living room or kitchen by a bed sheet hung from the ceiling. The main rooms were often decorated with photographs, religious sayings or statues, and a small stack of pots and pans used for cooking next to small gas or kerosene stoves and a plastic chairs.

Over the course of this research, we observed that hope is ubiquitous among our research population. However, we observed a gendered disparity in strategies engaged to achieve aspirations. Within households we found that while there was generally convergence in household level aspirations (i.e. both mothers, fathers, and children focused primarily on children being able to achieve success), there was divergence in observed strategies, or who engaged behaviors associated in the public health literature as risk behavior in order to achieve these aspirations. Often times, women, based on socio-cultural expectations and notions of sacrifices that need to be made for their children, engage in strategies that may lead to physical bodily harm in order to minimize harm to their kin. We observed that women, particularly mothers, often bore the burden of physical pain or diminished income to meet their individual needs. Below, we organize findings from our interviews and participant observation with mothers into three main interrelated themes: women’s physical embodiment of sacrifice; the justification for these strategies as redemptive; and the ways in which faith communities support women to cope with their perceived transgressions.

### Women’s physical embodiment of sacrifice

Strategies that we observed over the course of this research included activities that could result in greater exposure to potential physical or bodily harm (i.e. various forms of violence, sexually transmitted infections, stroke). The two most common strategies with potential physical consequences that we focus on in this paper were engagement in sex work and non-compliance with medical regimens for prevalent chronic conditions including hypertension, diabetes, and HIV.

Sex work, often times engaged on an ad hoc basis, was common across many of the women-headed households. Half of the women research participants readily admitted that in times of austerity they often had little economic choice but to engage in sex work. Many of the urban refugee women we worked with from various communities complained that they felt unprotected in the informal labor market and were often not paid as promised by their employers. This was especially challenging for Rwandan urban refugees, many of whom were about to lose or had been recently denied renewal of their refugee status under the purview of the Cessation Clause. For example, Bernadette, a 39 year old widow and mother of two young teenage boys confided:

- Well, you know, for us women, it isn’t like we want to do it (sex work). But with the way that people treat us refugees here, it can be hard to find work. So, if at the end of the month we were washing clothes or cleaning dishes for someone and they didn’t pay us what they promised, we can’t exactly go to the police and complain. So, you know, in order to be able to pay our rent, we become prostitutes. Even if it is just for a few days a month, but it helps us to eat, and to take care of everything else.

Interlinked with the theme of how strategies are justified (see next sub-section), among women-headed households sex work often eventually became sustained to enable household heads to support the children towards their aspirations for the future as edified by Elizabeth. Elizabeth, a 42 year old mother of two children in their 20s, explained that her husband had left to work in the Republic of Congo in 2016. A year later when she explained this, neither her nor her children had heard anything or received any financial support from him. As a woman who had never attended school, she explained to me that, “*je n’ai pas le choix, je dois faire ce que je dois faire, me prostituer, pour mes enfants*!” (I don’t have a choice, I have to do what I have to do, sell my body for sex, for my children).

Every household that we engaged with over the course of this research was struggling with at least one, and often multiple, chronic health conditions that required expensive and sustained medical follow-up including hypertension, diabetes, and HIV. From our observations, these conditions were more prevalent among adult women. Yet, mothers rarely talked about their health concerns with Yotebieng when discussing their aspirations or challenges they faced. This included Francoise, a 43 year old widow, who Yotebieng learned through her sons was living with diabetes and hypertension, but who only focused on what she was doing to ensure her two sons’ health and future livelihoods would not be affected by the slum conditions they were living in. When asked about how she managed her chronic health conditions, Francoise pointed out:

- You know, because of my living situation here, I recently fell gravely ill. But, you know, I am a mother, I can’t get ill. But anyhow, I know it is because of the poor living conditions that we are living in. I just couldn’t do anything about it because I needed to find money to continue to send my son to school. My other son is trying to get by in a store after finishing school each day. He even traveled to Bangui to work for a bit, and then came back and needed some assistance to start his own affairs here. And you know, that is of course my priority.

Discussion of ailing mothers and grandmothers engaging in strategies of sacrifice that included non-compliance to chronic health regimens was also articulated by other household members. Among several younger persons, we patterns of discussion around the challenge of continuing to support their aging mothers in ways that, as Jean Bosco articulated, “*nous empeche d’evoluer dans nos propres vies*” (holds us back from building our own lives). Further reiterating the sense of his grandmother’s necessary non-compliance, Jean-Bosco continued:

- It’s really complicated, I mean, just taking care of my own family (referring to wife and infant) is, but with grandmother, it is just impossible. But her illness worries me a lot. It isn’t a small illness, and it requires a lot of hospital visits. And when she gets sick, it is now me that has to spend my money, sometimes my boss needs to give it to me and cut it from my already meager salary … It is a huge burden (*fardeau*) on us.

Yet, all of this was in stark contrast to men heads of households. While men head of households shared aspirations related to the success of their children, over the course of this fieldwork we did not observe systematic non-compliance to medical regimens. On the contrary, we observed at times significant investments to improve their own health. Jean de Dieu, a 48 year old father who lived with his wife and two daughters, all as undocumented refugees from Rwanda, explained that they had closed their store and not been able to send their two young daughters to school because he spent over 400,000 FCFA ($800) on healthcare for himself. He had been struggling to try and figure out why his foot periodically swelled and was painful, and finding a solution to that was his priority. He clearly cared about his children and family in making his investment, however, the associated actions taken as a result were distinctly different. He justified his choices to invest in his health stating:

- I am the father of the house, so, you know, it is after we find a solution to my foot, after it is healed, that we will be better. My daughters are always crying to go to school, one day my older daughter even just showed up at the school near our house and tried to go in but the teacher turned her away. She cried the whole day! I think I am cured now, so hopefully we can re-open our store and I can finally send her to school. Now that I am cured.

This quote from Jean de Dieu illustrates a broader observation across our households: men and women took different strategies to support their children’s futures, with women often sacrificing bodily health, and men ensuring their own. These different strategies, however, were towards a convergent goal of ensuring success for their children as discussed below.

### Strategies as redemptive

These actions that are the focus of substantial public health literature on risk behavior, were in fact rational actions in the context of a precarious hope. Aspirations of mothers often centered on their children, and we observed women engaging in strategies in order to achieve their goals. Elizabeth (see above) felt forced to engage in sex work to provide for her children, and never spoke of the potential impact to her health or well-being, but rather, how the money she earned would help her son become a doctor so he could take care of her. Many of the women spoke of engaging in sex work temporarily as a way to provide for their households. If they did not admit directly that they were pushed to engage in this practice in the past, they told us about other women in the community that had to do it. Sex work was frequently employed as a way to get out of a current situation and improve the lives of children in the household by mothers. Lila, a 33-year-old mother of three young children, pulled Yotebieng aside one day when she met her at the store that Lila and her boyfriend worked at. She looked back over her shoulder to make sure her boyfriend was out of ear shot. She quietly clarified that she was willing to take any risk if it meant her two autistic children would have the chance of being “cured”:

- You know, it isn’t for me, or him (pointing towards her boyfriend with a head nod), but, for the kids (pause as she began crying) … I mean, with their autism, if they stay in Cameroon they will just not get the help they need … I would do anything I swear, anything, even sell myself, or get married to someone who just uses me for, you know …, but could take me to the United States, to be able to get them into one of those American schools. Those ones that can cure the kids.

Lila continued to explain to me that her “purpose” in life was to make sure her children succeeded, no matter what she had to do to achieve this.

Many women complained that it was difficult to treat chronic health conditions that they have, and that rather than becoming burdens on their children, they coped by reducing their ration and other expenses (including clothes, etc.). Four women who had acquired HIV via rape during the conflict in Rwanda, confided that they often skipped the medicine and clinical follow-up required of them if it meant that their growing children would have more resources to pursue their own education and professional objectives. Ivoire, despite being a pharmacist technician and knowing the potential deleterious health impacts of reducing the medicine she needed for her hypertension, readily skipped days or spit pills as a way to save for the education and medical follow-up for her daughters or grandson. Women took explicit steps to avoid getting in the way of their children’s futures, or “becoming a burden.” Jaqueline illustrated this in describing the adjustments she made in her life when she had children:

- When I first moved to Cameroon, I didn’t have children yet. And my husband was still here, and we had the means for me to think about my future. I was ambitious and wanted to open my own pharmacy. I was able to study and dream about what I would become. With my husband’s death, and one daughter pregnant, the other with heart failure, how am I supposed to even think about those things anymore? Of course I don’t take my medicines if that money can be used for something else, it is expensive!

Antoinette, an HIV positive and hypertensive older widow who was now living with one of her adult sons, his wife, and her three grandchildren, further elaborated the concern of feeling she was becoming a burden as a justification for medical non-compliance:

- The UNHCR wanted to restart my association (a group that aimed to bring together widowed women for economic empowerment), but you know, I realized it is only the UNHCR and their NGO (non-governmental organization) partners that benefit from it. They use our names, the list of all of our names, to have their funding. We were supposed to be helping older women like me support ourselves. Instead, I am a burden on my son. Us, older persons with chronic health conditions, we are expensive, I would rather him not have to pay for it anymore. And my son told me he is tired of paying for my transportation to go and beg the UNHCR to help me when they never come through anymore.

At times our research participants did feel the need to justify actions that they or others were taking, particularly when they spoke of sex work. They did this as many of these behaviors were frowned upon in their moral (religious and socio-cultural expectations of women) contexts. They would however often proceed to justify these as transgressions or sins that “God would forgive” since they were for a greater good. Indeed, in the conversations when we observed and asked about these strategies, women would turn the conversation to focus on the importance of avoiding the degradation of the situations of their husband or child if they did not undertake the activity. To the women we engaged with, the only “risk behavior” per se was inaction that could result in their husband or child not being able to achieve their self-defined objectives. The idea of serving a greater good allowed mothers to continue even when in their discourse they emphasized that they were morally conflicted.

### Faith communities and coping with perceived transgressions

All of the women who participated in this research were also devout Christians and regular church-goers. They justified the challenges involved in sex work and cutting their healthcare as a way of “God is testing us,” as Antoinette declared. Often we would observe religious ceremonies and prayers organized to directly confront and ask forgiveness for these strategies as observed during one of Yotebieng’s participant observations with Antoinette.

- Excerpt from Yotebieng’s field notes: Antoinette was sitting with her grandchildren eating when I found her. She told me that she was planning a mass on the 29th (the day I was leaving) at her other son’s house (as the son she lived with had converted to Islam), in memory of her husband, her son, and her sister who had all been killed. She looked down and explained to me that it was in ensuring the survival of the rest of her children that she had sometimes engaged in sex work. Or not taking her medicine even though she equated it to something that could be looked down on by God as she was “killing herself.” She explained to me that she hoped this mass would be able to ensure that their souls found where they “needed to go.” She also hoped her soul would find where it “needed to go” when it was her time. She seemed to be thinking a lot about death these days, and brought it up several times along with her ailing health. She gave me a hug and told me to pray for her.

Another example comes from Valerie, a single mother living with HIV who lacked any means to pay her rent and was unable to tap into her social networks for support. Her daughter came home and told her that she wanted to continue her education and pursue cosmetology. In order to ensure that her daughter could do this, and in hopes that this would pull them out of the informal settlement and dire poverty they had been living in, Valerie, despite her strong faith and conservative religious background, engaged in sex work in order to ensure the means that would pay for her daughter’s schooling. While sex work, she admitted, was not her ideal way of achieving her aspirations, it was with hope that these aspirations were achievable and that sex work was temporary that helped her accept it.

In this way, faith was a gendered double-edged sword. Their faith prescribed specific behaviors that were acceptable for women that they were not always able to adhere to, but on the other hand, faith also provided a means to cope with these transgressions in knowing that since they were temporary and forgivable. Over the course of this fieldwork we observed that mothers often engaged in decisions and work that they understood as “morally wrong,” “sins” or transgressions. However, this was always immediately followed by discussions of faith and the idea that everything was somehow God’s plan as a way to justify their decisions.

Marcelle, a single mother of one teenage daughter, illustrated the way that faith justified risks she took in skipping her antiretroviral medication:

- You see, where I am at, I can’t lie to you. We suffer! There is no way I can make enough money to support my daughter’s future just by cleaning clothes. I mean, I have rent, I have my HIV medicine, I have … so much, it just is overwhelming. So I know that there is always a market for women, and at the end of the month, when I need to, I supplement with sex work. I mean, guys pay me for it … But God, you know, he is really great! And he forgives! And with all of the money I am able to save, hopefully my girl and I, we can move to the United States and live dignified lives, at least her, I have already lost mine. But I have my faith, and God loves me. He will forgive me, and he has something better planned for us eventually. Otherwise he wouldn’t put me through this. I know God will help us. He won’t abandon us. There are moments where I ask myself if he will, but I know that our God can’t do that.

## Discussion

Given the well-documented gendered disparities in health and well-being outcomes across public health research, it is perhaps unsurprising that gender also affects underling risk-taking practices that may drive these disparities [56, 57]. Women, particularly mothers, are often the persons that engage the highest risks in order to ensure that their aspirations, hinging on the well-being of other household members, could be achieved [58]. Below we will discuss the implications of this research and the potential applications of this precarious hope framework. Overall, while this research focused on the case of Rwandan urban refugees in Yaoundé, we argue that our findings can also serve as an explanatory framework for other populations. For example, urban refugees in Cameroon, as well as in many parts of the world, are living alongside host populations that are living with similar socio-economic challenges. There is often much overlap in their broader risk environments. This framework takes those broader contexts into consideration, while also exploring the ways in which aspirations serve as motivating factors towards engaging in strategies, and the ways in which those aspirations and strategies are further informed and at times justified by socio-cultural expectations (including gender), political and economic factors, and household dynamics.

While several frameworks have been developed in public health to understand risk [4] and health behavior in its broader ecosocial context [2], including health behaviors around non-compliance to chronic disease regimens [1, 3, 10], the precarious hope framework explicitly includes an additional important motivating factor behind strategies engaged by our research participants: the future. The gendered nature of interlinked aspirations and strategies engaged is what led us to coin a new theoretical framework, one of precarious hope.

We argue that hope is a critical missing, yet often overlooked [59], component of health behavior that allows humanitarians and public health practitioners to contextualize and understand practices, including those which public health and humanitarian professionals categorize as “risky,” of the world’s marginalized. Aspirations stemming from this hope intersect with gender and wider geopolitical issues in terms of what kind of aspirations are feasible for different persons, and the unequal risks and sacrifices that different household members make to achieve these aspirations. Gendered divergence in behaviors engaged to achieve aspirations emerged as central to the framework of precarious hope. Hope and the associated aspirations and practices that stemmed from it were precarious for women given the many structural and socio-cultural expectations that served as barriers. As women’s aspirations often hinged on different household members, these strategies quickly emerged as a way that tried to minimize harm to specific household members, even if it meant potential harm to themselves. For example, our findings echoes other research that demonstrate how sex work across Africa in the context of high rates of violence and exposure to HIV is often linked with gendered poverty, structural violence, and women expressing the need to provide for their children [60–62]. This framework can also help us understand behavior that is considered a high risk behavior from a public health perspective in a way that allows us to understand the broader justification behind it, and potential barriers or opportunities to reducing potential public health harm.

This research also sheds light on the need to rethink risk behavior in the overall social, cultural, and geopolitical context in which risk behavior occurs. It has been argued that hope is not distributed equally [63, 64]. Intricately entwined and virtually inseparable from this unequal distribution of hope are the broader risk environments, and the ways in which these also lead to unequal distribution of risk behaviors. Zaloom [65] argues that in only focusing on risk behavior alone as an object of analysis, without situating it into a broader context of practice towards different long-term outcomes and the agency and constraints of the persons exercising their agency, social scientists obscure our ability to identify the ways in which these strategies can also be seen as productive by those who engage them.

Many of the observed strategies that we observed were linked to what would be coined non-compliance to the chronic health conditions that were ubiquitous among the Rwandan urban refugee community. There are demonstrable links between forced migration and increased vulnerability to chronic health conditions as the result of trauma, interrupted healthcare, and challenges in access to healthcare. This is particularly true in urban areas where services may not be subsidized as they are in camps or may be complicated by legal status issues, among other factors, are well documented in the scholarly literature [66–72]. Chronic conditions are often more prevalent among women, further illustrating risk in non-compliance linked with the nature of precarious hope [5–7].

Non-compliance to clinical regimens for non-communicable chronic diseases (NCD) was commonly observed among our research sample, and significant given the increasing burden of NCDs in urban areas globally. While a portion of public health studies on non-compliance interviewed patients to assess reasons for noncompliance in the patient’s own terms, such studies did not allow for elaboration and explanation of noncompliance by the patient in larger cultural terms [5–7]. Understanding these larger structural forces that form the root of the problem are critical to effectively improving patient compliance. The avoidance of becoming a burden justified an often gendered non-compliance to medical regimes for chronic illness. Faith and a hopeful, future orientation helped women to cope with the waiting associated with the achievement of their aspirations, and the physical suffering that many experienced (although rarely explicitly mentioned) as a result of some of the sacrifices they had to make.

Finally, this research underlines that strategies which in the public health field we qualify as “risky” are not understood the same way by those who engage these strategies [12, 73]. Few studies have acknowledged the insufficiency of identifying “high risk groups” that are more likely to engage in risk behavior, and have sought to assess local beliefs and practices that influence compliance to more effectively focus intervention policy [18, 19]. Our research further demonstrated the need to understand the socio-cultural and gendered expectations of the group which lead to these strategies (for example, women engaging in non-compliance to chronic health regimens to avoid becoming a burden on their kin).

In our research, reasons for engaging in strategies were complex and nuanced. When a person makes the decision to comply or not, he or she is influenced by cultural ideas about gender, moral implications, and economic constraints within the household and society at large. Awah et al. [74] pointed out that chronic disease patients adopt risk behavior including non-compliance as a way to manage their understanding of cure and treatment options provided by chronic disease therapies. In this research, while we observed women engaging in activities that could lead to their potential physical bodily harm, all of these actions were taken with direct links to broader aspirations and better futures. Studies that merely measure noncompliance within demographic or lifestyle variables can identify correlation, but provide no insight into the underlying causation for the associations they find. Drawing potentially false conclusions about policy based on an assumed causal relationship can lead to public health policy that many patients find to be irrelevant or not useful to them.

### Strengths and limitations

While our small sample size and qualitative design limits the generalizability of our findings, Yaoundé, Cameroon, represents a growing urban center in a lower-middle income country experiencing both demographic transition and increasing urbanization, allowing findings from this research to be applicable to other cities in Africa and the developing world. Furthermore, we acknowledge that our sample was heavily focused on the experiences of women from low-income households. The reasons for this were multifold. In regards to the positionality of the fieldworker (Yotebieng), a married woman which limited her access to many men in the community who did not always open up readily to her. Sensing this discomfort, as well as the discomfort from their partners or wives, Yotebieng did not want to insist, and many of her interactions with men in households occurred while their wives or daughters were present. While we cannot claim that the experiences of our research participants therefore represents all Rwandans in Cameroon, we argue that the situations and strong themes that emerged from this research are arguably reflective of populations, particularly women, living in informal settlements at the urban margins throughout the Central Africa region.

As a strength, qualitative research can provide insight into areas that we still know little about, with implications towards solutions, including engaging with policy makers and implementers on the unintended consequences of their policies and programs [75–77]. The long-term engagement involved in ethnographic approaches and the richness of data garnered from these repeated long-term engagements can lead to a discernment between what is said versus what is done. The implications of an ethnographic approach are powerful to understanding health behavior. Research participants would not often talk about some of the strategies that were socially unacceptable or could lead to poor health outcomes that they would take in order to achieve their aspirations in the first sitting. These strategies were something that we observed over time, or learned about in the context of our deeper conversations as we spent more and more time together. Trust was crucial to getting to the root of what our research participants usually referred to as transgressions. This allows for us to reframe the gaps and objectives to those of persons affected by humanitarian situations, rather than checklists of outsider perceived needs developed and applied across many contexts. Resources across the humanitarian sector are increasingly limited, as is an understanding of why some programs have worked well for some groups and not others across different contexts. We argue that failing to understand these contexts will lead to the development of programs that are destined to fail, and further undermine the dignity and agency of displaced persons, and that the long-term and contextually focused approach of ethnography offers a strong anecdote to this common issue in the humanitarian sector. This research enabled us to uncover a wide range of experiences within a group that is often lumped together into one “urban refugee” category, and the critical need to explore the particularities of a specific group. While introducing culture and aspects of “the particular” can complicate generalizations, it arguably can better inform policy makers and public health practitioners about the particular challenges and potential feasible solutions within their contexts.

Lastly, it is important to discuss the Yotebieng’s (the fieldworker’s) positionality in regards to the research participants. She, like many, who work with refugees, encountered a sense of heightened expectations. Despite clear informed consent and explanation of what the primary fieldworker could and could not offer, many research participants seemed to think that talking with her would bring them something. During her first trip to the field, her phone rang non-stop with refugees who wanted to meet her, confusing her current position as a researcher with her past experience in Cameroon and the Central Africa region as a public health professional and humanitarian. It became clear over the course of this research that no matter how many times one explain their research, its purpose, and the benefits, there is always hope hinging on telling one’s story to another person. This power-laden reality was one that Yotebieng continued to revisit and acknowledge over the course of her research in her field notes, and while some of these expectations appeared to diminish over time, given the long-term ethnographic approach to this research, she noted that it was important to continuously reflect on and ensure communication in regards to her positionality in the field.

### Policy and program implications

The precarious hope framework can be used to understand health behavior, particularly in the area of research in public health focused on “risk.” Understanding behaviors perceived from a public health or humanitarian perspective as “risky” from the perspective of the broader aspirational map of those engaging in these behaviors bears enormous applied potential. In understanding the rationale and justification for these “risks,” public health practice can aim to influence policy that better supports populations in the deficit areas of their lives that may lead them to engage in these “risks,” often times associated with strained access to capital or structures that allow them to provide adequately for their kin [12, 78]. Studying health behavior through a precarious hope framework allows us to identify the socio-cultural and political economic factors that may push people to engage in behaviors that have been correlated with harm or poor health outcomes, and devise strategies to mitigate or reduce the potential harm that may result from these strategies by addressing the driving factors.

No one knows better how to survive adverse circumstances better than those who are living through it [29, 79]. Furthermore, as Bulhan [80] poignantly underlines, it is time for more bottom-up approaches, rather than the top-down strategies of international agencies which may serve to further disempower and hamper the aspirations of the worlds marginalized. Furthermore, these top-down approaches have been largely unsuccessful to date, despite the enormous resources poured into them [81]. A large meta-analyses on focused psychosocial interventions in humanitarian contexts also underlines the importance of maintaining hope for the overall health and well-being of displaced persons [82]. But it is imperative that this hope is understood from the aspirations and corresponding practices and agency of populations who are affected by the particular problem being studied [82].

The precarious hope framework allows public health professionals to understand how individuals living at the world’s margins think past their immediate hardship, develop aspirations for their future, and take action to achieve these goals. Through their stories and experiences, we can identify the seeds of promising and achievable means through which we can support “risk-reduction programs.” [78] On the other hand, we can learn from their experiences and aspirations about what policy changes, programs, or activities may have inadvertently forced persons to engage in practices that they otherwise would have preferred to avoid [78]. Lastly, understanding health behavior from this perspective can allow for messages to reduce these practices to be devised, acknowledging the reasons for these practices, and using culturally appropriate language and alternatives to these practices [78]. This also means identifying the policies and programs which may inadvertently undermine the well-being of household members, particularly women.

## Conclusions

The findings underline the importance of conceptualizing and understanding how aspirations are prioritized at the household level and the strategies different household members, particularly women, take contingent on cultural and social norms and available capital, prior to developing risk-reduction programs. In the humanitarian sector, many of the services provided are determined and distributed at the household level. Our evidence underlines the importance in exploring what is hidden in the household and needs to be understood and uncovered in order to ensure that the well-being of all household members is supported through social services. These household dynamics, often times gendered, can then be brought to focus for effective policy and social service programs that ensure the well-being of all household members [83]. Our framework of precarious hope offers a solution in underlining the need to pay attention to these often gendered household dynamics and the differential effects of changing social service frameworks and humanitarian policy on household members.

In understanding the rationale and justification for risk behavior, public health practice can aim to influence policy that better supports women in the areas of their lives that may lead them to engage in these strategies, often times associated with ensuring better futures for their kin [78]. Through our proposed framework of precarious hope, we first aimed to understand the specific contexts in which aspirations were shaped and decisions made, including what would be perceived from a public health or humanitarian perspective as risk behavior by mothers. In the context of increasingly limited options associated with impending legal precarity and the challenges in accessing livelihoods, healthcare, and education associated with that, we found that these gendered strategies needed to be reframed and understood as a way that women chose to take control and create possibilities for their kin. In this way, household level approaches should also focus on supporting these kin, so that mothers do not have to engage in strategies that may negatively affect their health and well-being.

Our research suggests that risk behavior needs to be considered, and mitigated, through a future-oriented understanding of aspirations, gendered household dynamics, and other contextual considerations that may often go overlooked. Strategies engaged by research participants need to be understood as a social, and not individual, level practice, as posited in existing ecosocial frameworks of health behavior [2–4]. Our framework posits that aspirations and their links with access to various forms of capital and socio-cultural expectations of women are key motivating factors that need to be included in these broader frameworks. Eggerman and Panter-Brick [84] emphasize a bourgeoning and important area of research which they coin an “anthropology of hope.” [79]We argue that within this framework, hope can be used to understand risk behavior. Indeed, using a framework that focuses on hope allows us to better understand the aspirations and related practices that emerge in the context of displacement, opening the potential in understanding the ways in which refugees endure and actively work to overcome hardship, and the potential harms and justifications from the perspectives of those engaging in these strategies.

We posit an important relationship between aspirations, hope, and peaceful cohabitation between the growing number of urban refugees living in cities in developing countries and their host-communities. This is especially crucial to explore now, as we have seen increases in violent extremist activities in Cameroon and across West and Central Africa. Through the lens of an anthropology of precarious hope, we situate the driving forces behind strategies and hope as intricately entwined, and necessary to be considered together including the ways in which socio-cultural gendered realities may exacerbate disparities in health behavior. In this way we can understand why and when risk behaviors are taken, in turn providing us with fuel to advocate for policy changes that address the underlying impetus for risk behavior which may not be on the radar of health and humanitarian policy makers.

## Abbreviations

CWS: Church World Services
*Francs CFA*: FCFA Central Africa Economic Region currency
HIV: Human Immunodeficiency Virus
NCD: Non-Communicable Diseases
NGO: Non-Governmental Organization
UNHCR: United Nations High Commissioner for Refugees
